# Determinants of Poor Sleep Quality During the COVID-19 Pandemic Among Women Attending Antenatal Care Services at the Health Facilities of Debre Berhan Town, Ethiopia: An Institutional-Based Cross-Sectional Study

**DOI:** 10.3389/fpsyt.2022.841097

**Published:** 2022-03-18

**Authors:** Nakachew Sewnet Amare, Basazinew Chekol, Agazhe Aemro

**Affiliations:** ^1^Department of Midwifery, Debre Berhan University, Debre Birhan, Ethiopia; ^2^Department of Anesthesiology, College of Medicine and Health Science, Debre Tabor University, Debre Tabor, Ethiopia; ^3^Department of Medical Nursing, College of Medicine and Health Science, University of Gondar, Gondar, Ethiopia

**Keywords:** COVID-19, sleep quality, women, Ethiopia, pregnancy

## Abstract

**Background:**

Women’s ability to get sleep can be affected by pregnancy-related hormonal changes or other external stressful situations like the coronavirus disease 2019 (COVID-19).

**Objective:**

The objective of this study was to assess the proportion of poor sleep quality during the COVID-19 pandemic and its determinants among pregnant women attending antenatal care (ANC) services.

**Methods:**

An institutional-based cross-sectional study was conducted among 423 women attending ANC services at the health facilities in Debre Berhan Town, Ethiopia, from May to June 2020. A systematic random sampling technique was used to select the required samples. The tool consisted of questions that assessed (1) socio-demographic characteristics, obstetric and health care service-related characteristics; and media exposure to get information regarding COVID-19 infection; (2) To assess sleep quality; the Pittsburgh Sleep Quality Index (PSQI) was applied. And a global score of >5 indicates poor sleep quality, and a global score of ≤5 indicates good sleep quality.

**Result:**

The overall prevalence of poor sleep quality was 62.8%, and was associated with pregnant women aged ≥46 years (AOR = 4.27), being in the third trimester (AOR = 2.51), being multigravida (AOR = 2.72), and having co-morbidity (AOR = 3.57).

**Conclusion:**

The prevalence of poor sleep quality among pregnant women during the pandemic was found to be high. Advanced maternal age, third trimester pregnancy, being multigravida, and having comorbidity were determinants of poor sleep quality among pregnant women during the COVID-19 pandemic.

## Background

Pregnancy is a process accompanied by dramatic hormonal changes (1–3) that create significant anatomical, physiological, and biochemical changes in a woman’s life. These hormonal changes can also have profound effects on women’s ability to sleep and may result in poor sleep quality (4–7). Sleep is a physiological process and a basic requirement for the physical and mental well-being of pregnant women and their fetuses (2, 8). Furthermore, sleep disturbances and short sleep duration are common during pregnancy (9, 10), which can have an impact on health-related quality of life. Although a sleep problem may start soon after conception, it worsens in frequency and duration as the pregnancy advances more (11).

Sleep has a critical role in promoting the health of both the mother and the fetus (12). Worldwide, approximately one-third of all pregnant women reported that they had disturbed sleep during pregnancy (13). Disturbed sleep during pregnancy is linked to several complications, including preeclampsia, pre-mature birth, gestational diabetes, postpartum depression, and intrauterine growth retardation (9, 13). Studies in different areas of the world reported that the prevalence of poor sleep quality among pregnant women was 17% in Peru (14), 41.2% in Vietnam (15), 43.1% in the Asian population (4), 45.7% in Canada (16), 51.8–87% in China (10, 11), 53.3% in Pakistan (17), 59.5% in Indonesia (18), 73% in the United States (1). Some studies showed the prevalence of poor sleep quality among pregnant women before the pandemic in Ethiopia at Jimma medical center 30.8% (8) and Wadila primary hospital 68.4% (2).

The determinants of poor sleep quality among pregnant women significantly varied with the trimester of the pregnancy. Overall, poor sleep quality had a direct correlation with advanced age, maternal education level, being unmarried, anxiety, depression, stress, gestational age, multiparous, multigravida, and watching television in the bedroom (2, 8, 10, 11, 15, 18). In addition, there are also many external stressful situations, like the coronavirus disease 2019 (COVID-19), which can result in disturbed sleep during pregnancy.

Studies reported that COVID-19 harmed sleep quality in the general populations (19–21). This problem may become worsen in pregnant women. The global pandemic COVID-19 infection has been shown to have an important impact on pregnant women and their fetuses. Pregnant women may experience fear of contracting COVID-19 (22) and its consequences, which can result in anxiety, depression, stress, and insomnia (23, 24). Additionally, pregnant women encountered partial immune suppression and became more susceptible to COVID-19 infection during the pandemic (25). All of this can lead to the development of negative emotions and cause pregnant women to be more concerned about the COVID-19 infection, resulting in sleep disruption (12) and poor sleep quality (26).

COVID-19 related morbidity and mortality are shockingly increasing in Ethiopia. This phenomenon may create stressful situations, especially for those at high risk, like pregnant women. Consequently, this COVID-19-related negative emotion among pregnant women could result in difficulty in getting sleep. However, there are limited studies done regarding sleep quality among pregnant women during the COVID-19 pandemic in Ethiopia. So, this study aimed to assess the prevalence of poor sleep quality and its determinant factors among pregnant women attending antenatal care (ANC) services during the era of the COVID-19 pandemic at Debre Berhan Town, Ethiopia in 2020.

## Materials and Methods

### Participants and Procedure

An institutional-based cross-sectional study was conducted from May 1 to June 1, 2020, in Debre Berhan Town public health institutions. The source populations for the study were all pregnant women who are attending antenatal care services in Debre Berhan town. All pregnant women who are attending antenatal care services in the Town during the study period and fulfill the inclusion criteria were included as the study population. The sample size was determined by using the single population proportion formula with the assumption of 50% poor sleep quality, a 95% confidence interval, and a 5% marginal error. After adding a 10% non-response rate, the final sample size was 423. In this study, pregnant women who visited the public health institutions in Debre Berhan Town for ANC services were included in the study. And pregnant women who were unable to communicate effectively due to serious illness were excluded from the study. To select our study participants, all public health facilities in Debre Berhan town were considered, and then based on the number of pregnant women that visited the public health facilities during the preceding month before data collection, proportional allocation of the total sample size was carried out to get the required sample from each public health facility. Finally, the determined samples were selected with a mean age of 28 years (SD ± 4.86) by a systematic random sampling technique.

### Data Collection Tools and Procedures

Pretested and interviewer-administered questionnaires were used for the whole survey. The tool consisted of 33 items categorized in to two sections, (1) socio-demographic characteristics, obstetric and health care service-related characteristics; and media exposure to get information regarding COVID-19 infection with a total of 14 items; (2) items to assess sleep quality by the Pittsburgh Sleep Quality Index (PSQI). The Pittsburgh Sleep Quality Index contains 19 Likert-type and open-ended questions. Respondents were asked about their overall sleep quality and how frequently they had experienced certain sleep difficulties in the previous month. The 19 items were combined to form seven component scores, each of which had a range of 0–3, with a higher score indicating more acute sleep disturbances. Then, the seven component scores were added to yield a single global score ranging from 0 to 21, with the higher score indicating severe sleep difficulties in all areas. PSQI developers have suggested a cutoff score of 5 for the global scale as it was 88.5% valid to correctly identify the problem (27–29). The Cronbach alpha of PSQI in the current study was 0.72. Furthermore, the data was collected by trained BSc midwives, and the consistency and completeness of the data were checked daily by supervisors.

### Variable of Interest

#### Sleep Quality

Is defined based on the PSQI score; hence, a global score of >5 indicates poor sleep quality, and a global score of ≤5 indicates good sleep quality (27).

#### Exposure to the Media

Women who had access to either television, radio, or read newspapers at least once a week was considered exposed to the media.

#### Co-morbid Disease

Is defined as the co-existence of diagnosed chronic medical conditions like asthma, diabetes mellitus, heart disease, hypertension, depression, cancer, and chronic kidney disease among pregnant women (30).

### Statistical Analysis

The data was first entered into EPI INFO™ 7 and then exported to STATA version 14, statistical software for analysis. Frequencies and cross-tabulations were applied to summarize descriptive statistics of the data, and tables were used for data presentation. A binary logistic regression model was used to identify factors associated with poor sleep quality. Those variables with a *p*-value less than or equal to 0.2 from the bi-variable analysis were candidates for multivariable analysis. Variables with a *p*-value of less than 0.05 in multivariable analysis were declared as statistically significant factors for poor sleep quality. Moreover, the association was measured using odds ratios with a 95% confidence interval. Model fitness was also checked by the Hosmer-Lemeshow goodness of fit test (*P*-value = 0.491).

### Ethics Approval and Consent to Participate

This study was approved by the Institutional Review Board (IRB) of Debre Berhan University and an official permission letter was gained from the concerned body. Written informed consent was obtained from each participant before conducting the actual data collection process. Additionally, confidentiality was maintained by avoiding registration of personal identifiers and no raw data was given to anyone other than the investigator.

## Results

### Pregnant Women’s Socio-Demographic, Obstetric, and Healthcare-Related Characteristics

Of the 423 pregnant women, almost all (99.8%) participated in the analysis. Of these, 175 (41.47%) were found under the category of age 25 years or less, with a mean age of 28 years (SD ± 4.86). The majority (86.73%) of the participants were married, and 164 (38.86%) of the women had primary education levels. Of all the participants included in the analysis, 241 (57.11%) were housewives. About one-third (34.36%) of the participants’ husbands were at the primary education level, and 216 (51.18%) of their husbands were merchants. One hundred ninety-four (46%) pregnant women lived 2–5 km away from the health facility. Similarly, more than half (54.5%) of the participants were in the third trimester and 250 (59.24%) were multi-gravida. On the other hand, the majority of the women (89.8%) had no known co-morbid diseases. Around 81.5 and 57.4% of the women watched television and heard the radio to get information, including COVID-19, respectively. But, the majority of the women (90.52%) didn’t have the habit of reading newspapers during the era of the pandemic (Table 1).

**TABLE 1:** The socio-demographic and obstetric-related characteristics of pregnant women attending antenatal care (ANC) services in Debre Berhan (*n* = 422).

Variable	Categories	Frequency	Percentage (%)
Maternal age	<25 years	175	41.47
	26–35 years	67	15.88
	36–45 years	108	25.59
	≥46 years	72	17.06
Marital status	Single	27	6.40
	Married	366	86.73
	Divorced	29	6.87
Maternal education	No formal education	138	32.70
	1^0^ education	164	38.86
	2^0^ education	59	13.98
	Diploma and above	61	14.45
Maternal occupation	Housewife	241	57.11
	Government employee	73	17.30
	Private employee	60	14.22
	Student	48	11.37
Husband education	No formal education	94	22.27
	1^0^ education	145	34.36
	2^0^ education	94	22.27
	Diploma and above	89	21.09
Husband occupation	Employed	163	38.63
	Merchant	216	51.18
	Daily labor	43	10.19
Number of persons living with her	≤4 individual	339	80.33
	>4 individuals	83	19.67
Distance to the health facility	<2 km	192	45.50
	2 – 5 km	194	45.97
	>5 km	36	8.53
Trimester of pregnancy	1st trimester	63	14.93
	2nd trimester	129	30.57
	3rd trimester	230	54.50
Gravidity	Primigravida	172	40.76
	Multigravida	250	59.24
Co-morbid disease	Present	43	10.19
	Absent	379	89.81
Watch TV before bedtime	Yes	344	81.52
	No	78	18.48
Heard the radio before bedtime	Yes	242	57.35
	No	180	42.65
Ever read a newspaper about COVID-19	Yes	40	9.48
	No	382	90.52

### The Prevalence of Poor Sleep Quality Among Pregnant Women

**FIGURE 1:**
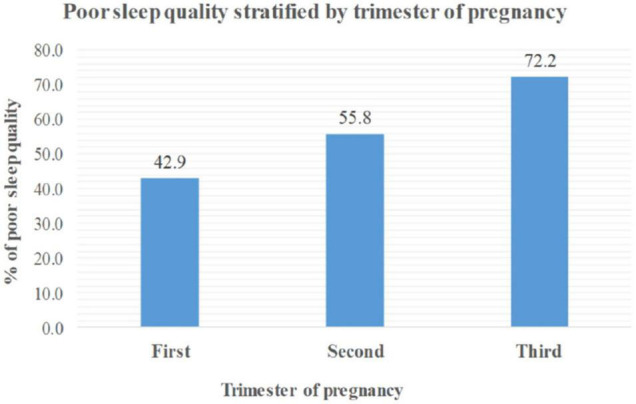
Poor sleep quality among pregnant women stratified by trimester at Debre Berhan town, Ethiopia, 2020.

The overall prevalence of poor sleep quality among pregnant women attending ANC service at health facilities in Debre Berhan town was 62.8% (95% CI: 58.1–67.3). The prevalence of poor sleep quality among pregnant women increases with gestational age, showing the highest proportion in the third trimester (Figure 1).

### Determinants of Poor Sleep Quality Among Pregnant Women

After applying multivariable binary logistic regression, four variables, namely maternal age, trimester of pregnancy, gravidity, and presence of co-morbidity, were significantly associated with poor sleep quality among pregnant women. Thus, the odds of having poor sleep quality among pregnant women aged ≥46 years was 4.27 times that of women aged less than 25 years [AOR = 4.27; 95% CI: 1.43–12.79]. Similarly, the likelihood of experiencing poor sleep quality was 2.51 times higher in the third trimester compared to the first trimester [AOR = 2.51; 95% CI: 1.175–42]. Likewise, multigravida women were 2.72 times more likely than primigravida women to have poor sleep quality [AOR = 2.72; 95% CI: 1.34–5.50]. Pregnant women with the co-morbid disease were 3.57 times more likely to have poor sleep quality than pregnant women without comorbidity [AOR = 3.57; 95% CI: 1.45–8.78] (Table 2).

**TABLE 2:** Bivariate and multivariate sleep quality analysis among pregnant women in Debre Berhan (*n* = 422).

Variable	Category	Sleep quality		COR (95%CI)	AOR (95%CI)	*P*-value
		Good	Poor			
Maternal age	<25 years	88	87	1.00	1.00	
	26–35 years	17	50	2.97 (1.59–5.56)*	1.91 (0.77–4.72)	0.160
	36–45 years	40	68	1.72 (1.05–2.81)*	1.28 (0.61–2.70)	0.519
	≥46 years	12	60	5.06 (2.54–10.05)*	4.27 (1.43–12.79)	0.009**
Marital status	Single	9	18	1.00	1.00	
	Married	143	223	0.78 (0.34–1.78)	0.42 (0.13–1.34)	0.142
	Divorced	5	24	2.39 (0.69–8.39)*	2.14 (0.46–9.91)	0.333
Maternal education	No formal education	44	94	1.04 (0.55–1.98)	2.28 (0.94–5.52)	0.067
	1^0^ education	69	95	0.67 (0.36–1.25)*	1.36 (0.59–3.13)	0.476
	2^0^ education	24	35	0.71 (0.34–1.49)	1.78 (0.66–4.77)	0.254
	Diploma and above	20	41	1.00	1.00	
Maternal occupation	Housewife	97	144	1.00	1.00	
	Government employee	23	50	1.46 (0.84–2.56)*	1.19 (0.56–2.53)	0.651
	Private employee	14	46	2.21 (1.15–4.25)*	2.03 (0.92–4.48)	0.079
	Student	23	25	0.73 (0.39–1.36)	2.18 (0.81–5.81)	0.121
Husband education	No formal education	36	58	0.74 (0.40–1.36)	0.92 (0.34–2.47)	0.872
	1^0^ education	58	87	0.69 (0.39–1.20)*	0.88 (0.38–2.07)	0.773
	2^0^ education	35	59	0.77 (0.42–1.43)	0.87 (0.33–2.26)	0.769
	Diploma and above	28	61	1.00	1.00	
Husband occupation	Employed	58	105	1.00	1.00	
	Merchant	77	139	0.99 (0.65–1.54)	1.21 (0.57–2.57)	0.615
	Daily labor	22	21	0.53 (0.27–1.04)*	0.56 (0.20–1.56)	0.269
No. of people living with	≤4 individual	132	207	1.00	1.00	
	>4 individuals	25	58	1.48 (0.88–2.48)*	0.58 (0.29–1.16)	0.125
Distance to the health facility	<2 km	78	114	1.00	1.00	
	2 – 5 km	66	128	1.33 (0.88–2.01)*	0.92 (0.52–1.63)	0.780
	>5 km	13	23	1.21 (0.58–2.53)	0.46 (0.15–1.39)	0.171
Trimester of pregnancy	1st trimester	36	27	1.00	1.00	
	2nd trimester	57	72	1.68 (0.92–3.09)*	1.31 (0.55–3.14)	0.542
	3rd trimester	64	166	3.46 (1.94–6.15)*	2.51 (1.17–5.42)	0.019**
Gravidity	Primigravida	91	81	1.00	1.00	
	Multigravida	66	184	3.13 (2.08–4.72)*	2.72 (1.34–5.50)	0.006**
Co-morbid disease	Present	7	36	3.37 (1.46–7.77)*	3.57 (1.45–8.78)	0.006**
	Absent	150	229	1.00	1.00	
Watch TV before bedtime	Yes	123	221	1.39 (0.84–2.29)*	1.85 (0.79–4.36)	0.158
	No	34	44	1.00	1.00	
Heard the radio before bedtime	Yes	83	159	1.34 (0.89–1.99)*	1.12 (0.59–2.12)	0.713
	No	74	106	1.00	1.00	
Read newspaper about COVID-19	Yes	16	24	0.88 (0.45–1.71)		
	No	141	241	1.00		

*Candidate variables for multivariate analysis at p-value ≤ 0.2. **Statistically significant factors at a p-value of <0.05.

## Discussion

In this study, the overall magnitude of poor sleep quality among pregnant women during the era of COVID-19 was 62.8% (95% CI: 58.1–67.3). This is in line with studies conducted in Indonesia (59.5%) (18) and Northern Ethiopia (68.4%) (2). But, the magnitude of poor sleep quality in the current study was lower than in studies conducted in Turkey (88%) (26), China (87%) (11), and the United States (73%) (1). The discrepancy might be due to differences in socio-demographic characteristics and in the time when the study was conducted. On the other hand, poor sleep quality in the current study was higher than in studies from Peru, China, Pakistan, Vietnam, the Asian population, and Canada, ranging from 17 to 53.3% (4, 10, 14–17, 31, 32). The majority of these previous studies were conducted before the outbreak of the global COVID-19 pandemic, so the women couldn’t worry about the infection compared to the women in the current study. Furthermore, being quarantined and apart from loved ones during the COVID-19 pandemic might increase the proportion of depressive symptoms, stress, and anxiety. These psychological situations in turn affect the sleep quality of pregnant women (18, 33).

The odds of having poor sleep quality were 4.27 times higher in pregnant women aged 46 years and older than in women aged less than 25 years. This is supported by studies from China (11), and Ethiopia (2, 8). As maternal age increases, the likelihood of women being affected by physiological and psychological factors will also increase (34). This might in turn result in poor sleep quality (35).

Similarly, a significant association between gestational age and sleep quality was detected, showing that sleep quality declines as pregnancy advances. Hence, the risk of developing poor sleep quality among pregnant women in the third trimester was 2.51 times greater than that of pregnant women in the first trimester. This is consistent with studies from China (10, 11), Turkey (36), and Ethiopia (2). As gestational age increases, the tendency for frequent urination, even at night, increases and results in disturbed sleep (37). Additionally, when the woman approaches her end date of delivery, she might worry about childbirth, finance, labor, and delivery, or the baby’s health, which all could be risk factors for disturbed sleep (37, 38). Weight gain, along with hormonal and physiological changes, induces pregnant women to have sleep-disordered breathing like snoring and obstructive sleep apnea, which in turn disturbs the normal sleep pattern (38).

A significant association between gravidity and sleep quality was discovered, showing that sleep quality declines as the number of pregnancies increases. When compared to primigravida, the likelihood of having poor sleep quality was 2.72 times among women with multigravida. This is consistent with research on pregnant women in Jimma, Ethiopia (8), and South Korea (39). This may be explained by the fact that maternal sleep quality is disturbed as a result of being overstressed about bearing extra roles after childbirth and the way they integrate the new role into their pre-existing responsibilities. Excessive worry caused by rehearsing physical pain during labor and delivery may also contribute to poor sleep quality in multigravida women (39).

Lastly, the presence of co-morbid disease among pregnant women was found to be significantly associated with poor sleep quality. Hence, pregnant women with co-morbid disease were 3.57 times more likely to develop poor sleep quality compared to pregnant women without comorbidity. This is supported by studies from Indonesia and the United States which declared that depression and gestational diabetes mellitus were directly associated with poor sleep quality (1, 18), respectively. This could be a reason for extra worry among pregnant women with medical comorbidity, which might induce a disturbed sleep pattern. Additionally, fear of a bad outcome for her baby and her as a result of existing comorbidity may contribute to the occurrence of poor sleep quality.

## Conclusion

Nearly two-thirds of pregnant women in this study had poor sleep quality. Women with advanced age, being in the third trimester, multigravida, and comorbidity were associated with poor sleep quality among pregnant women. Giving special attention to women of advanced age, third trimester pregnancy, multigravida, and counseling of the women with comorbidity in their consecutive antenatal care visits is crucial to reduce the risk of developing poor sleep quality and its consequences.

## Data Availability Statement

The raw data supporting the conclusions of this article will be made available by the authors, without undue reservation.

## Ethics Statement

The studies involving human participants were reviewed and approved by the Debre Berhan University College of Health Science research committee. The patients/participants provided their written informed consent to participate in this study.

## Author Contributions

NA conceived the idea, designed the work, and collected the data. BC collected the data and participated in the manuscript writing. AA analyzed the data, interpreted the results, and was a major contributor in writing the manuscript. All authors read and approved the final manuscript.

## Conflict of Interest

The authors declare that the research was conducted in the absence of any commercial or financial relationships that could be construed as a potential conflict of interest.

## Publisher’s Note

All claims expressed in this article are solely those of the authors and do not necessarily represent those of their affiliated organizations, or those of the publisher, the editors and the reviewers. Any product that may be evaluated in this article, or claim that may be made by its manufacturer, is not guaranteed or endorsed by the publisher.
